# Bioinformatics Approaches for Fetal DNA Fraction Estimation in Noninvasive Prenatal Testing

**DOI:** 10.3390/ijms18020453

**Published:** 2017-02-20

**Authors:** Xianlu Laura Peng, Peiyong Jiang

**Affiliations:** 1 Li Ka Shing Institute of Health Sciences, The Chinese University of Hong Kong, Hong Kong, China; laurapeng@link.cuhk.edu.hk; 2 Department of Chemical Pathology, The Chinese University of Hong Kong, Prince of Wales Hospital, Hong Kong, China

**Keywords:** noninvasive prenatal testing, circulating cell-free DNA, fetal DNA fraction

## Abstract

The discovery of cell-free fetal DNA molecules in plasma of pregnant women has created a paradigm shift in noninvasive prenatal testing (NIPT). Circulating cell-free DNA in maternal plasma has been increasingly recognized as an important proxy to detect fetal abnormalities in a noninvasive manner. A variety of approaches for NIPT using next-generation sequencing have been developed, which have been rapidly transforming clinical practices nowadays. In such approaches, the fetal DNA fraction is a pivotal parameter governing the overall performance and guaranteeing the proper clinical interpretation of testing results. In this review, we describe the current bioinformatics approaches developed for estimating the fetal DNA fraction and discuss their pros and cons.

## 1. Introduction

The discovery of circulating cell-free fetal DNA in maternal plasma [1] has created a paradigm shift in noninvasive prenatal testing (NIPT), which has rapidly made its way into clinical practices worldwide, for example, cell-free DNA-based chromosomal aneuploidy detection [2,3,4,5,6,7,8] and diagnosis of monogenic diseases [9,10,11,12,13,14,15]. The circulating cell-free DNA (cfDNA) in a pregnant woman is a mixture of predominant maternal DNA derived from the hematopoietic system of the mother [16,17] and fetal DNA released through the apoptosis of cytotrophoblast cells during fetal development [18,19]. The proportion of fetal DNA molecules among the total cfDNA molecules in maternal circulation is expressed as fetal DNA fraction, which is a paramount factor for determining the overall performance of NIPT [15,20,21,22] and interpreting clinical assessments [7,23,24,25].

In noninvasive fetal aneuploidy detection, the fetal DNA fraction in maternal plasma is linearly correlated with the extent of chromosomal abnormalities present in plasma of pregnant women [3,6,7]. The fetal DNA concentration below 4% in a maternal plasma sample would suggest a potential issue present in the quality control (QC) step, because the limited amount of fetal DNA molecules to be detected and analyzed may give rise to a false negative result [20,26,27,28]. Therefore, it is important to estimate the fetal DNA fraction accurately, making sure that it has passed the QC threshold to guarantee a sufficient amount of fetal DNA present in a testing sample and make it possible to arrive at a proper interpretation of the sequencing result. In addition, the fetal DNA fraction has been incorporated into bioinformatics diagnostic algorithms by a number of laboratories [7,23,24].

Monogenic diseases comprise a larger proportion of genetic diseases than chromosomal aneuploidies [15]. However, the cfDNA-based NIPT for single-gene diseases is much more challenging, because the cfDNA in maternal plasma is generally of minor population, hampering the reliable deduction of the maternal inherence of fetus at single-nucleotide resolution. Technologically, the development of relative haplotype dosage analysis (RHDO), which utilizes information regarding parental haplotypes flanking the variants of interest, has been demonstrated to greatly improve the accuracy of single-gene disorder detection [9,10,13]. More recently, researchers have illustrated that the use of linked-read sequencing technology allows for directly ascertaining parental haplotypes surrounding the genes of interest, making RHDO analysis a universal NIPT method for single-gene diseases [29]. This work has made an important step forward towards the real clinical utility regarding cfDNA-based single-gene disease testing. Such RHDO analysis took advantage of the fetal DNA fraction as a key parameter to determine the statistical thresholds, indicating if a particular maternal haplotype presumably inherited by the fetus exhibits a statistically significant over-presentation in maternal plasma of a pregnant woman [9,23].

**Figure 1:**
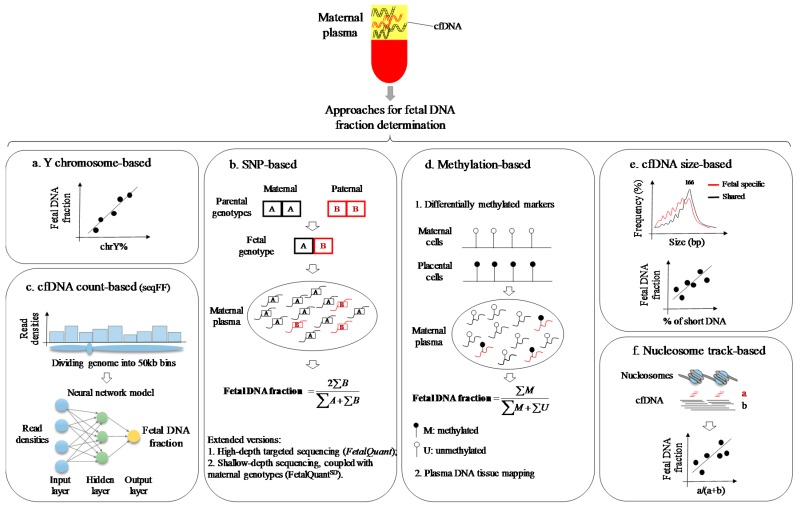
Schematic illustration of current approaches for the determination of fetal DNA fraction in maternal circulating cell-free DNA (cfDNA). (**a**) Y chromosomal (chr) sequence-based fetal DNA fraction estimate [3,22]; (**b**) Single-nucleotide polymorphism (SNP)-based approach. A direct way to estimate the fetal DNA fraction is to use the SNP loci, where both mother and father are homozygous but with different alleles. The resulting fetal genotype is obligately heterozygous. In maternal plasma, the fetal DNA fraction can be directly deduced by calculating the proportion of fetal specific alleles [9,30]. Based on this concept, two extended versions of SNP-based methods for fetal DNA fraction estimate have been developed, namely *FetalQuant* and FetalQuant^SD^, which can be used without the need of both paternal and maternal genotype information [31,32]; (**c**) cfDNA count-based approach. Read densities across the genome-wide 50 KB windows are fitted into a neural network model to predict the fetal DNA fraction [33]; (**d**) Differential methylation-based approaches [17,26,34,35]; (**e**) cfDNA size-based approach. The proportion of short cfDNA molecules is correlated with fetal DNA fraction [36]; (**f**) Nucleosome track-based approach. Cell-free DNA distribution at the nucleosomal core and linker regions is correlated with fetal DNA fraction [37].

In this review, we discuss a number of existing approaches for the determination of fetal DNA fraction, as well as their advantages and disadvantages (Table 1). The simplified principles for these approaches are diagrammatically depicted in Figure 1.

## 2. Current Approaches Developed to Estimate Fetal DNA Fraction

### 2.1. Y Chromosome-Based Approach

In the early works, genetic markers located on Y chromosome which are paternally inherited, such as gene *SRY*, *DYS14* and *ZFY*, were used to indicate the fraction of fetal DNA molecules based on PCR assays [23,38,39]. For instance, the ratio of the concentration of the sequences from Y chromosome to that of an autosome was used for the determination of fetal DNA fraction. In the context of NIPT using massively parallel sequencing, the proportion of all sequence reads from Y chromosome can be translated to the fetal DNA fraction [3,22]. Although these methods are simple and accurate, they are only applicable to pregnancies carrying male fetuses.

### 2.2. Maternal Plasma DNA Sequencing Data with Parental Genotype-Based Approach

With the use of parental genotypes, fetal-specific alleles in maternal plasma can be readily identified from the sequence reads. Briefly, the fetal genotypes are obligately heterozygous at single-nucleotide polymorphism (SNP) loci, where both father and mother are homozygous but with different genotypes (e.g., A/A for paternal genotype and C/C for maternal genotype). Then the fetal DNA fraction can be quantified by calculating the ratio of fetal-specific alleles (A) to the total alleles in plasma DNA [7,9,30,40]. Even though this method is a direct and accurate way to assess the fetal DNA fraction and generally considered as a gold standard [9], the feasibility of this approach is sometimes hindered by the requirement of parental genotypes, because (1) only maternal blood samples would be collected and maternal plasma DNA are subject to sequence for NIPT in most clinical settings; and (2) it is not uncommon that the genotype of the biological father may not be available in practice [41].

### 2.3. High-Depth Sequencing Data of Maternal Plasma DNA-Based Approach

To obviate the requirement of parental genotype information, an approach called *FetalQuant* was developed to measure the fetal DNA fraction through the analysis of maternal plasma DNA sequencing data at high depth using targeted massively parallel sequencing [31]. In this method, a binomial mixture model was employed to fit the observed allelic counts with the use of the underlying four types of maternal-fetal genotype combinations (AA_AA_, AA_AB_, AB_AA_, AB_AB_, where the main text and subscript represent the maternal and fetal genotypes, respectively). In this model, the fetal fraction was determined through the maximum likelihood estimation. The predicted result of this method is very close to the one deduced by the parental genotypes-based approach (the correlation coefficient is not available). However, the limitation of this approach would be that the sequencing depth is required to be as high as ~120× by targeted sequencing to robustly determine the fetal alleles [31].

### 2.4. Shallow-Depth Maternal Plasma DNA Sequencing Data with Maternal Genotype-Based Approach

As an extended version of *FetalQuant*, FetalQuant^SD^ [32] was recently developed based on shallow-depth sequencing data coupled with only maternal genotype information. The rationale of this approach is to take advantage of the fact that any alternative allele (non-maternal alleles) present at an SNP locus where the mother is homozygous would theoretically suggest a fetal-specific DNA allele. Briefly, the homozygous sites in a pregnant woman were identified by genotyping her blood cells using microarray technologies. Then, plasma DNA molecules with alleles different from the maternal homozygous sites (i.e., non-maternal alleles) were identified, which were specifically derived from the father in theory. Thus, the fractions of such non-maternal alleles were hypothesized to correlate with fetal DNA fractions under the assumption that the error rates stemmed from sequencing and genotyping platforms are relatively constant across different cases. Therefore, a linear regression model was first trained between the fraction of non-maternal alleles and actual fetal DNA fraction estimated by parental genotypes-based approach, and then the fetal DNA fractions were predicted with the use of the trained model in an independent validation dataset, exhibiting a very high accuracy (*r* = 0.9950, *p* < 0.0001, Pearson correlation) even using 1 million sequencing reads. However, the parameters in this model might be varied according to sequencing and genotyping platforms, because various platforms are characterized with different error properties, which may contribute to the measured non-maternal alleles. On the other hand, the extent of heterozygosity might be different in different ethnic groups, which could confound the accuracy of fetal DNA fraction prediction. The advantage of this model is that once the final well-trained model is achieved, it could be readily applied to any datasets, as long as they are generated from the same platform and population.

### 2.5. Shallow-Depth Maternal Plasma DNA Sequencing Data-Based Approach

Recently, a new approach, named SeqFF, has been developed, attempting to make it possible to directly estimate fetal DNA fraction from the routine data of NIPT without any additional effort. In this approach, using single-end random sequencing of the maternal plasma, read count within each 50 KB autosomal region was analyzed to fit a high-dimensional regression model [33]. The normalized read counts in 50 KB bins originating from chromosomes except chromosomes 13, 18, 21, X, and Y were used as predictor variables, and the model coefficients were determined by making use of elastic net (Enet) and reduced-rank regression model [33]. SeqFF showed a good correlation with Y chromosome-based method in two independent cohorts (*r* = 0.932 and 0.938, respectively, Pearson correlation) [33]. However, such high-dimensional model would require large-scale samples during training, and the performance appeared to be greatly deteriorated when the fetal DNA fraction is below 5%, possibly because the number of cases with fetal DNA fraction <5% was not sufficient to train the Enet model.

### 2.6. Fetal Methylation Marker-Based Approach

DNA methylation is a process by which a methyl group is added to cytosine nucleotides [42,43]. In mammalian somatic cells, the DNA methylation of cytosine in CpG dinucleotides is frequently methylated (~70% of the CpGs) [44]. Different organs have been suggested to show variable methylation profiles, which would allow us to identify the tissue of origin analyzing the regions with differential methylation states [17,45]. Indeed, researchers used the placenta-specific methylation markers to estimate the fetal DNA concentration [26,34]. For example, a methylation-sensitive restriction enzyme has been used to digest hypomethylated maternal-derived *RASSF1A* promoter sequences, while it left the methylated counterparts of the fetal-derived sequences unaffected, thus allowing the discrimination of the methylated fetal DNA molecules from the unmethylated maternal background for the calculation of fetal DNA fraction [34]. Similarly, based on five differentially methylated regions comparing placental tissue and maternal buffy coat mined by using methyl-cytosine immunoprecipitation and CpG island microarrays, Nygren et al. developed a fetal quantitative assay (FQA) permitting the calculation of fetal DNA fraction in a plasma sample [26]. In FQA, by measuring the copy number of total DNA (maternal and fetal) and fetal methylated DNA after methylation-sensitive restriction enzyme digestion, the assay achieved good agreement with Y chromosome-based quantification (*r* = 0.85, *p* < 0.001, Pearson correlation). However, the analytical process used for quantifying these epigenetic markers involves digestion with methylation-sensitive restriction enzymes, and thus its stability needs to be further verified in large-scale datasets generated from different research centers.

Furthermore, massively parallel bisulfite sequencing provides an alternative way to estimate the fetal DNA fraction according to the ratio of fetal-derived DNA molecules within differentially methylated regions [35]. Using such bisulfite sequencing, the placenta has been demonstrated to exhibit a different methylation profile compared with other tissues [17,35]. Therefore, a general approach, referred to as plasma DNA tissue mapping, for disentangling tissue contributors to cell-free DNA has been developed by leveraging the principle that different tissues within the body show different DNA methylation patterns. Using whole-genome bisulfite sequencing, the methylation profile of cell-free DNA across over 5800 DNA methylation markers was used to correlate the tissue-related methylation profiles, for the inference of the proportional contributions from different tissues in plasma [17]. Using this new approach, placenta contribution was verified by genotype-based approaches. However, this genome-wide bisulfite sequencing-based tissue mapping algorithm in the present version would be too expensive for routine NIPT.

### 2.7. Cell-Free DNA Size-Based Approach 

Fetal-derived and maternal-derived DNA molecules in a plasma sample have been observed to exhibit different fragmentation patterns, namely, fetal DNA being generally shorter than maternal DNA [9,46]. Therefore, a higher fetal DNA fraction should be theoretically associated with an increased percentage of short DNA molecules. Using paired-end sequencing, Yu et al. developed a new method to estimate fetal DNA concentration based on the ratio between the count of fragments ranging from 100 to 150 bp and from 163 to 169 bp [36]. These size cutoffs gave their optimal performance among multiple size combinations. In the training dataset consisting of 36 samples, a linear regression model was established between the size ratio and fetal DNA concentration determined by the proportion of chromosome Y sequences (*r* = 0.827, *p* < 0.0001). Then using the derived model, the size ratio was translated to the fetal DNA fraction for each sample in the validation dataset. Intriguingly, the authors also proposed to calculate the size ratio using capillary electrophoresis of sequencing libraries directly, which is readily available before sequencing without additional costs.

### 2.8. Cell-Free DNA Nucleosome Track-Based Approach

Recently, the investigation of nucleosomal origin of plasma DNA has been increasingly recognized as an appealing direction, which has been discussed in a number of studies [9,36,37,47]. One important clue directing to such origin has been unravelled in two studies with the use of the high-resolution size profiling of maternal plasma DNA [9,36]. It has been reported that the size distribution of the total maternal plasma DNA is characterized by a 166 bp major peak with a series of small peaks occurring at 10 bp periodicities, suggesting that a predominant population of plasma DNA molecules have a size of 166 bp. In contrast, fetal DNA molecules were found to have a dominant population with 143 bp in size. It has been speculated that the 166 bp molecules would represent cfDNA containing the nucleosome core plus the linker [9]. However, the 143 bp molecules would suggest molecules subject to the trimming of linker DNA [9]. On the basis of this hypothetical model, Straver et al. pooled maternal plasma DNA from 298 cases to generate a hypothetical “nucleosome track” [37]. Interestingly, the frequency of reads starting within 73 bp upstream and downstream regions of the inferential center of nucleosome was found to be positively correlated with the fetal DNA fraction, however, giving a relatively lower correlation coefficient than other methods (*r* = 0.636, *p* = 1.61 × 10^−18^, Pearson correlation). Thus, further development of a “nucleosome track”-based approach is needed for the clinical requirement.

## 3. Conclusions

The past decade has witnessed a tremendous advance in the technologies and bioinformatics algorithms for the analysis of circulating cfDNA. With the availability of massively parallel sequencing, noninvasive prenatal testing has become increasingly popular and presented itself as an exemplar in translational medicine research. In NIPT, a rapid, simple, accurate and cost-effective way to estimate fetal DNA fraction is highly desired, typically for the endeavors to make NIPT for single-gene diseases clinically practical. In particular, the accuracy of the estimation of low fetal DNA fraction is essential for determining the QC states and interpreting the clinical outcomes. On the other hand, the fetal DNA fraction could be related to pregnancy outcome; for example, the low fetal DNA fraction may be associated with small or dysfunctional placentas [48], suggesting its potential diagnostic value. Therefore, a large-scale validation for the accuracy of low-fetal DNA fraction estimation would still be needed for some aforementioned approaches, for example, size-, count- and nucleosome profile-based methodologies. We may expect that further in-depth analyses for such properties regarding size and nucleosome profiles would shed new insights into the mechanisms of cell-free DNA generation. As reported in the latest ultra-deep plasma DNA study [49], it was revealed that a number of preferred DNA ends in maternal plasma carry information directing to their tissue of origin (fetal- or maternal-derived DNA). The ratio of the number of fetal-preferred ends to maternal-preferred ends is positively correlated with the fetal DNA fraction in maternal plasma [49]. This novel direction of cfDNA exploration regarding fragment ends has opened up new possibilities to study the complexity associated with non-randomness of plasma DNA ends, providing a new way to investigate the highly orchestrated cfDNA fragmentation patterns. More studies are needed to elucidate the relationship between the various factors as well as their interactions, for example, methylation [17], nucleosome footprints [47], and the underlying mechanisms governing the end-cutting patterns of plasma DNA. More studies in such new directions will lead to a better understanding toward the principles of fetal DNA generation, as well as the factors governing the fetal DNA fraction in different physiological and pathological conditions.

## Figures and Tables

**Table 1:** The summary of current approaches for estimating fetal DNA fraction.

Approaches	Advantages	Limitations
Y Chromosome [3,22]	Simple and accurate	NOT applicable for pregnancies with female fetuses
Maternal plasma DNA sequencing data with parental genotypes [9,30]	Direct and accurate	Paternal DNA may not be available
Targeted sequencing of maternal plasma DNA (*FetalQuant)* [31]	Sequencing maternal plasma DNA only; accurate	High sequencing depth is required
Shallow-depth sequencing of maternal plasma DNA coupled with maternal genotypes (FetalQuant^SD^) [32]	Shallow-depth sequencing of maternal plasma DNA; accurate	Maternal genotype requirement will add additional costs; the recalibration curve is required to be rebuilt for different sequencing and genotyping platforms
Shallow-depth maternal plasma DNA sequencing data (SeqFF) [33]	Only shallow-depth sequencing of maternal plasma DNA; single-end sequencing; easy to be integrated into the routine noninvasive prenatal testing (NIPT)	Large-scale samples are needed to train the neutral network; need to improve the accuracy when the fetal DNA fraction is below 5%
Differantial methylation [17,26,34,35]	Accurate	Either bisulfite conversion or digestion with methylation-sensitive restriction enzymes may affect the accuracy; genome-wide bisulfite sequencing is too expensive and prohibitive for the routine NIPT
cfDNA fragment size [36]	Only shallow-depth sequencing of maternal plasma DNA; easy to be integrated into the routine NIPT	Moderate accuracy; paired-end sequencing would increase the costs
Nucleosome track [37]	Only shallow-depth sequencing of maternal plasma DNA	Lower accuracy; high-depth sequencing data is required during the training step
